# Double-Hybrid Density Functional Theory for Core Excitations: Theory and Benchmark Calculations

**DOI:** 10.1021/acs.jctc.2c01222

**Published:** 2023-02-01

**Authors:** Dávid Mester, Mihály Kállay

**Affiliations:** † Department of Physical Chemistry and Materials Science, Faculty of Chemical Technology and Biotechnology, Budapest University of Technology and Economics, Müegyetem rkp. 3, H-1111Budapest, Hungary; ‡ ELKH-BME Quantum Chemistry Research Group, Müegyetem rkp. 3, H-1111Budapest, Hungary; ¶ MTA-BME Lendület Quantum Chemistry Research Group, Müegyetem rkp. 3, H-1111Budapest, Hungary

## Abstract

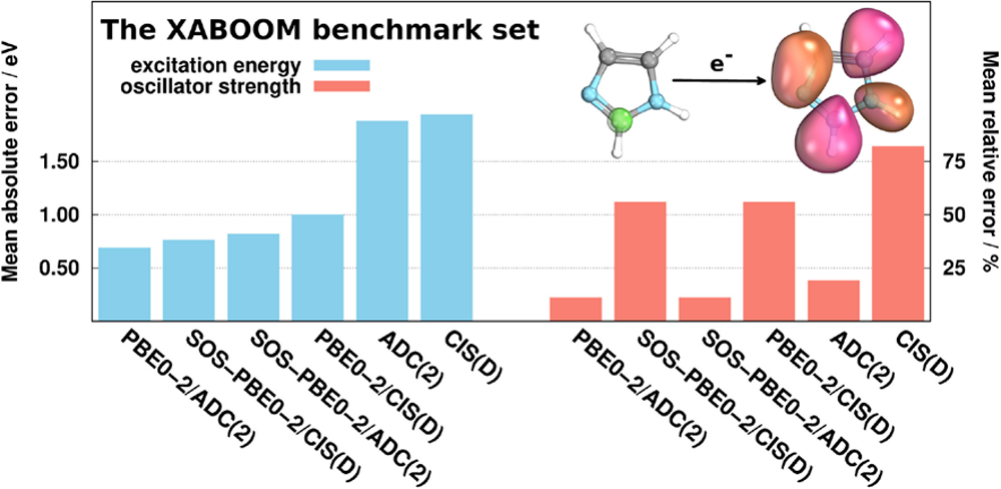

The double-hybrid (DH) time-dependent density functional theory is extended to core excitations. Two different DH formalisms are presented utilizing the core–valence separation (CVS) approximation. First, a CVS-DH variant is introduced relying on the genuine perturbative second-order correction, while an iterative analogue is also presented using our second-order algebraic-diagrammatic construction [ADC(2)]-based DH ansatz. The performance of the new approaches is tested for the most popular DH functionals using the recently proposed XABOOM [*J. Chem. Theory Comput.***2021**, 17, 1618] benchmark set. In order to make a careful comparison, the accuracy and precision of the methods are also inspected. Our results show that the genuine approaches are highly competitive with the more advanced CVS-ADC(2)-based methods if only excitation energies are required. In contrast, as expected, significant differences are observed in oscillator strengths; however, the precision is acceptable for the genuine functionals as well. Concerning the performance of the CVS-DH approaches, the PBE0-2/CVS-ADC(2) functional is superior, while its spin-opposite-scaled variant is also recommended as a cost-effective alternative. For these approaches, significant improvements are realized in the error measures compared with the popular CVS-ADC(2) method.

## 1 Introduction

In recent times, significant advances have taken place in modern experimental instruments, such as synchrotrons and radiation sources of free-electron lasers.^1,2^ These developments have turned X-ray spectroscopy into one of the main characterization techniques in many fields, for example, surface science, biochemistry, and organic electronics.^3−6^ The key to success is that these techniques provide chemical fingerprints and detailed information about the chemical structure of the compounds due to the localized and characteristic nature of core orbitals. Theoretical tools play a crucial role in the analysis of the resulting complex experimental spectra. Consequently, the development of efficient approaches for core-excitations is currently an actively researched area of modern quantum chemistry.

To calculate excitation spectra, iterative diagonalization schemes are used typically, which yield the energetically lowest eigenvalues.^7,8^ As core-excited states are located in the high-energy X-ray region of the spectrum, such calculations for extended systems would be highly inefficient using standard procedures because all underlying valence-excited states should be calculated as well. Invoking the core–valence separation (CVS) approximation,^9^ the couplings between core- and valence-excited states can be neglected *a priori*. The basic idea of the CVS approximation relies on the significant energetic and spatial separation between core and valence occupied orbitals. Consequently, these two blocks of the Hamiltonian can be easily decoupled. In this case, the equations are solved only for the targeted core-excited spaces, and this simple trick also provides notable savings in computation time. We note that other techniques are also available to tackle the high-energy region of the spectra, such as the restricted-window or energy-specific schemes.^10−12^

The algebraic-diagrammatic construction (ADC) formalism^13^ was elaborated through the diagrammatic perturbation expansion of the polarization propagator and the Møller–Plesset (MP) partitioning of the Hamiltonian. Its second-order variant [ADC(2)]^14,15^ is a well-established and popular approach for valence-excited calculations as it offers an appropriate compromise between accuracy and computation time.^16^ The CVS approximation was first combined with the ADC scheme in the 1980s.^9,17^ The scope of CVS-ADC(2) has been significantly extended by the pioneering work of Dreuw and co-workers.^18−22^ Its performance was comprehensively tested for core excitations, and a good agreement between the calculations and experiments was revealed.^18,20,23,24^ The systematically improvable CVS-ADC formalism enables the indirect inclusion of orbital relaxation effects via couplings to higher-excited configurations. The CVS-ADC(2) method is rigorously derived from the second-order MP (MP2) theory, and its secular matrix elements in the doubles–doubles block are expanded only in the zeroth order. In the extended variant [CVS-ADC(2)-x],^25^ which is an ad hoc extension of the strict approach, the doubles block is treated up to the first order. This inclusion increases the accuracy of the calculations,^20,23,26^ although it also makes the method more expensive.

Thirty years after the initial CVS studies, the approximation was also extended to coupled cluster (CC)^27^ and multireference^28^ methods. A simple and easy-to-implement eigenvalue solver was proposed by Coriani and Koch,^27^ which allows us to target core excitations for any existing excited-state CC code. However, this scheme contains unnecessary operations as the matrix elements are projected out after their evaluation. Later, an effective implementation for the frozen-core equation-of-motion CC singles and doubles (fc-CVS-EOM-CCSD) was also presented by Coriani and co-workers,^29^ where the CVS approximation was fully utilized. Due to its excellent results and performance, the method quickly became popular in the community and is considered as a standard approach for medium-sized molecules.^30−36^ It is also noteworthy that the CVS approximation was combined with the emerging similarity-transformed EOM-CCSD method,^37^ while implementations for higher-order EOM-CC approaches, including triple excitations, were also presented by Matthews et al.^38,39^

Nowadays, time-dependent (TD) density functional theory (DFT) is the method of choice for extended molecular systems as its computational costs are relatively low.^40−42^ The formalism was extended to core excitations by Stener and co-workers,^43^ and its performance was comprehensively discussed in excellent reviews.^23,44−46^ It is well-known that DFT-based methods generally suffer from self-interaction error (SIE), which corresponds to the approximate treatment of the exchange-correlation (XC) potential. This problem also leads to a strong underestimation of the energies of core-excited states. Accordingly, tailoring XC functionals for core excitations is still an active field of research.^47−51^ Over the years, various approaches have been developed to remedy the SIE problem in DFT. One of the most notable attempts in this direction is the double-hybrid (DH) theory.^52^ In its genuine excited-state variant,^53^ a hybrid TDDFT calculation is performed, and subsequently, a second-order contribution is added *a posteriori* to the excitation energy, relying on the configuration interaction singles (CIS)^54^ with a perturbative second-order correction [CIS(D)]^55^ method. Taking into account their computational costs, it is clear that one should expect some overhead in comparison with the hybrid TDDFT methods; however, it cannot be expected that the CIS(D) correction will become the bottleneck for practical applications. The accuracy and efficiency of DH functionals for valence-excited states have been demonstrated in numerous studies, and their superiority to conventional DFT methods has been proven;^56−64^ however, their performance for core excitations is an entirely unexplored field. We note that other promising or well-established approaches have also been elaborated to improve DFT results for core excitations, such as the orthogonality constrained DFT,^65^ constricted variational DFT,^66,67^ real-time TDDFT,^68^ short-range corrected TDDFT,^47^ transition-potential DFT,^69,70^ orbital-optimized DFT,^71−74^ and the restricted open-shell Kohn–Sham (KS)^75−77^ approches.

In this paper, we combine the CVS approximation with the DH theory. First, we present a CVS-CIS(D)-based formalism for genuine DH-TDDFT functionals. Thereafter, a more advanced ansatz is also elaborated, combining the CVS-ADC(2) method and our ADC(2)-based DH formalism.^78^ The performance of the most popular DH functionals is benchmarked against fc-CVS-EOM-CCSD results using the recently proposed XABOOM^24^ test set. The errors in excitation energies and oscillator strengths are assessed in detail for various types of excitations, and the outcomes regarding accuracy and precision are also discussed.

## 2 Theory

### 2.1 CIS(D)-Based CVS-DH Formalism

In the most common formalism of DH theory,^52^ the ground-state XC energy is obtained as
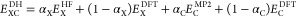
1where *E*_X_^DFT^ and *E*_C_^DFT^ are the semilocal exchange and correlation energies, respectively, while *E*_X_^HF^ stands for the exact Hartree–Fock (HF) exchange energy and *E*_C_^MP2^ is the MP2 correlation energy. The ratio of the corresponding contributions is handled by adjustable mixing factors denoted by α_X_ and α_C_. In this two-step scheme, the calculations start with solving the self-consistent KS equations, including the HF exchange contribution and the DFT exchange and correlation potentials. Thereafter, a perturbative MP2-like correction evaluated on the KS orbitals is added to the XC energy. The flexibility of the energy functional can be increased using spin-scaling techniques.^79,80^ In these cases, the same- and opposite-spin contributions to the MP2 correlation energy are scaled separately, and a more accurate description of the ground-state properties can be achieved.^63^ The excitation energy is also obtained in two steps in the genuine extension of DH theory for excited-state calculations.^53^ That is, the first step of the calculations is to solve a Hermitian eigenvalue equation relying on the Tamm–Dancoff approximation (TDA),^81^ and then a perturbative second-order correction is added to the excitation energy.

The combination of the above approach and the CVS approximation is fairly straightforward; however, to the best of our knowledge, no attempt had been made previously in this direction. Accordingly, the corresponding equations are briefly presented. Similar to the original scheme, in the first step, one has to solve a symmetric eigenvalue equation as
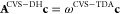
2where **A**^CVS–DH^ denotes the modified Jacobian, **c** is the singles excitation vector, and ω^CVS–TDA^ is the TDA excitation energy within the CVS approximation. The elements of the modified Jacobian^43,44^ are written as
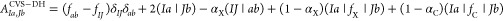
3where *f*_*pq*_ stands for a corresponding Fock-matrix element and (*Ia*|*Jb*) is a four-center electron repulsion integral in the Mulliken convention. The notation follows the convention that *I*, *J*... and *i*, *j*... are the active (core) and inactive occupied molecular orbital indices, respectively, while *a*, *b*... and *p*, *q*... denote virtual and general orbitals, respectively. The DFT exchange term is given by
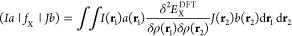
4where ρ(**r**) stands for the electron density, and **r** is a vector for electron coordinates. A similar expression holds for the DFT correlation.

Having the solution of eq 2 at hand, a perturbative second-order correction is calculated using the single excitation coefficients and transition energy obtained. As the CIS(D) approach^55^ is regarded as one of the natural excited-state extensions of the MP2 method, its usage seems to be a plausible choice for this purpose. Accordingly, the final DH excitation energy within the CVS approximation is obtained as
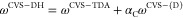
5where ω^CVS–(D)^ is the second-order correction. Note that spin-scaling techniques can be applied to excited-state DH calculations as well,^82−85^ and their use is also straightforward within the CVS approximation. The CIS(D) method has already been employed for core excitations to improve the poor performance of CVS-CIS.^44,86^ However, the CVS approximation, which significantly simplifies the working equations, was not used in these studies for the second-order correction. Utilizing the restriction that the single and double excitation vectors have to hold elements only with one active index, the perturbative correction is calculated as
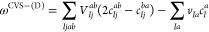
6In this expression, the intermediate *v*_*Ia*_ contains the contribution of the corresponding ground-state amplitudes, and its elements read explicitly as
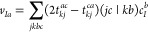
7where *t*_*ij*_^*ab*^ stands for a MP2 doubles amplitude, which is given as
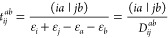
8with ε_*p*_ as the corresponding orbital energy. The double excitation coefficients within the CVS approximation are obtained as
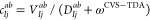
9using the intermediate *V*_*Ij*_^*ab*^, which is written as
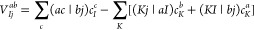
10

As the perturbative correction is only an energy correction for the CIS(D)-based DH methods, the calculation of the oscillator strengths is fairly straightforward, and similar expressions can be used as for hybrid TDDFT calculations. That is, invoking the CVS approximation, the transition density matrix needed for the ground to excited state transition moments can be expressed as
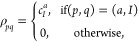
11and the oscillator strength (*f*) in the length gauge can be defined by
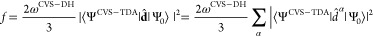
12where Ψ^CVS–TDA^ and Ψ_0_ denote the excited- and ground-state wave function, respectively, while the sum of squares on the right-hand side is the dipole strength and **d̂** = (*d̂*^*x*^, *d̂*^*y*^, *d̂*^*z*^) stands for the dipole operator. The *x* component of the transition electric dipole moment is obtained as
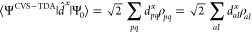
13where *d*_*pq*_^*x*^ stands for the *x* component of the dipole moment integrals in the molecular orbital basis.

As discussed in refs (44) and (86), the CIS(D) results are closer to the experiment than the CVS-CIS and hybrid CVS-TDDFT values; however, the excitation energies for Rydberg states, which are out of scope in this study, are significantly underestimated, and qualitatively incorrect spectra were obtained for particular systems. A similar performance can be assumed for the CVS-CIS(D) method as the CVS approximation has no effect on these outcomes. It will be interesting to see if the CVS-DH theory can further improve the results, at least for spectral properties.

### 2.2 ADC(2)-Based CVS-DH Formalism

The CVS-ADC(2) method^9,18^ is a popular approach in the community. The iterative fifth-order scaling method offers an appropriate compromise between accuracy and computational costs. As the algorithmic considerations are similar to the original ADC(2) approach, after slight modifications, an existing ADC(2) code can also calculate core excitations. The working equations of the method are briefly presented herein; for the interested readers, we recommend the work of Dreuw et al.^18,20,21^ In practice, a nonlinear eigenvalue equation is solved. The corresponding sigma vector is obtained as
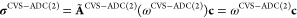
14where **Ã**^CVS–ADC(2)^ is the so-called effective Jacobian and ω^CVS–ADC(2)^ denotes the ADC(2) excitation energy within the CVS approximation. The Jacobian matrix can be split into two parts as
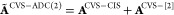
15where **A**^CVS–CIS^ is the CVS-CIS Jacobian, and all of the terms including second-order contributions are collected into matrix **A**^CVS–[2]^. Of course, a similar decomposition can also be made for the sigma vector:
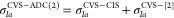
16The elements of the CVS-CIS sigma vector read explicitly as
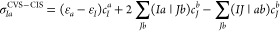
17while the second-order contributions within the CVS approximation are calculated as
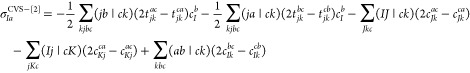
18

For the simplified elements of the transition density matrix, a linear-response CC consistent formalism was proposed by Köhn and co-workers.^87,88^ The explicit expressions using the CVS approximation are given for its various blocks by
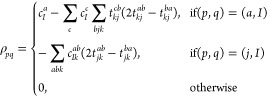
19The oscillator strength is calculated similarly to eqs 12 and 13, replacing the corresponding quantities with those obtained from CVS-ADC(2).

As ADC(2) can also be regarded as one of the excited-state analogues of the MP2 method, similar to the CIS(D) approach, an ADC(2)-based DH formalism can be derived as well.^78^ In this case, an ADC(2)-like calculation is performed with a modified effective Jacobian where the CIS Jacobian is replaced by the DH Jacobian, and the second-order terms are scaled by an empirical factor. This modification can also be carried out within the CVS approximation using eq 15. Thus, the resulting effective Jacobian reads as
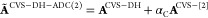
20where **A**^CVS–DH^ is defined by eq 3. In practice, the corresponding DFT contributions must be added to the sigma vector, and the second-order terms defined by eq 18 are scaled by α_C_.

The benefits of the ADC(2)-based formalism compared with the CIS(D) analogue were discussed in detail in refs (78) and (89). Accordingly, we now focus only on the most significant differences between the ADC(2) and CIS(D) approaches. In addition, extensive comparison for core excitations have not been made so far; therefore, the following findings are strictly valid only for valence-excited states. First, concerning transition energies, ADC(2) moderately but consistently outperforms CIS(D);^16^ thus, an improvement in the calculated transition energies is expected. This observation can be explained by the fact that, in the case of CIS(D), the doubles correction is added *a posteriori* to the CIS excitation energy, while these excitations are treated iteratively for the ADC(2) method. This improvement is especially noticeable when the double excitation contributions are significant. Second, in contrast to CIS(D), the ADC(2) method allows us to evaluate the transition moments at a higher level taking into account the effect of double excitations. Therefore, a higher quality of the computed oscillator strengths is predicted.

## 3 Computational Details

### 3.1 Calculation of the Numerical Results

The new approaches have been implemented in the Mrcc suite of quantum chemical programs and will be available in the next release of the package.^90,91^Mrcc was utilized in all of the calculations as well. In this study, Dunning’s correlation consistent basis sets (cc-pV*X*Z, where *X* = D and T)^92,93^ and their diffuse function augmented variants (aug-cc-pV*X*Z)^94^ were used. The density-fitting approximation was utilized in both the ground- and excited-state calculations, and the corresponding auxiliary basis sets of Weigend and co-workers^95−97^ were employed for this purpose. The conventional CVS spaces were used for most of the molecules, while the exceptions, where a so-called tailored space was applied, will be discussed in the following subsection.

For the DFT contributions, the exchange and correlation functionals of Perdew, Burke, and Ernzerhof (PBE),^98^ Becke’s 1988 exchange functional (B88),^99^ the correlation functional of Lee, Yang, and Parr (LYP),^100^ and Perdew’s 1986 correlation functional (P86)^101^ were used. The built-in functionals of the Mrcc package were employed in all cases.

The errors utilized to evaluate the excitation energies are calculated by subtracting the reference values from the computed ones. In this study, the accuracy and precision of the methods are also assessed. Thus, the main statistical error measures are the mean absolute error (MAE), standard deviation (SD), and maximum absolute error (MAX). The SD is obtained as
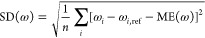
21where the mean error (ME) is given as
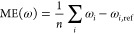
22while the MAX is simply taken as
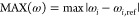
23In addition, further error measures, such as ME, error span, and root-mean-square error are also included in the Supporting Information; however, their discussion will be omitted. For the oscillator strengths, the relative error of the intensities is in focus. Accordingly, in this case, the SD is given as
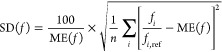
24where the ME is defined as
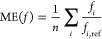
25To benchmark the accuracy of the methods in this regard, the mean relative error (MRE) is used. This measure is calculated as
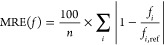
26All the computed excitation energies and oscillator strengths are also available in the Supporting Information.

### 3.2 XABOOM Benchmark Set

For the benchmark calculations, the recently proposed XABOOM^24^ test set was selected, which contains 40, primarily organic compounds. The selection of the molecular systems was inspired by the well-known benchmark set of Thiel and co-workers.^102^ Consequently, the compilation contains unsaturated aliphatic hydrocarbons, heterocycles, aromatic hydrocarbons, carbonyl compounds, nucleobases, etc. The test set only incorporates 1*s* → π* transitions as the main interest from experimental aspects. The benzoquinone molecule is excluded in our benchmark calculations as the HF reference used in ref (24) is unstable. Therefore, the inspected excitations comprise 71 carbon, 21 nitrogen, and 22 oxygen *K*-edge transitions.

The original study uses fc-CVS-EOM-CCSD and ADC(2)-x results as a reference. This reasonable choice is easy to understand considering the computational costs of the methods. As the XABOOM benchmark set contains molecules of up to 18 atoms, the calculations using higher-order methods would be unfeasible. On top of that, spectral properties, such as oscillator strengths, are not available for such approaches.^38,39^ The performance of the reference methods has been extensively tested against experimental measurements with great success;^20,23,30,36,103^ unfortunately, the comparisons against higher-order methods are very limited. The performance of CVS-EOM-CCSD was tested by Matthews et al.^38,39^ using CC methods including triple excitations as a reference. As shown, the excitation energies are systematically overestimated by around 1.5 eV. However, their benchmark compilation contains only small molecules of up to 2 heavy atoms, and most of the inspected excitations were Rydberg states. Accordingly, the performance of the fc-CVS-EOM-CCSD and ADC(2)-x methods for the XABOOM test set is still hard to judge. Nevertheless, if the proposed DH functionals approach the accuracy or precision provided by the reference methods, they could be a reasonable alternative at a significantly lower computational cost.

In this study, mainly the fc-CVS-EOM-CCSD results are considered as a reference; however, the error measures calculated from the ADC(2)-x results are also presented in the Supporting Information. This choice can be explained by the fact that it avoids the bias caused by the similarity in the formalism of the CVS-ADC(2) and CVS-ADC(2)-x methods. The reference values were obtained with the so-called aT/T/D basis set. It contains aug-cc-pVTZ basis sets for atoms directly involved in double or triple bonds, cc-pVTZ basis sets for the remaining non-hydrogen atoms, and cc-pVDZ basis sets for hydrogens. As discussed in the original paper, this basis set is a highly appropriate choice for 1*s* → π* excitations. The conventional CVS spaces were used during the calculations, except for the C *K*-edge transitions of imidazole, pyridine, pyridazine, cytosine, uracil, and adenine molecules. In these cases, a so-called tailored CVS space was applied to avoid the mixing of the states. For convenience, hereinafter, the CVS prefix will be dropped from the name of the approaches.

### 3.3 Assessed Methods

In this study, the most popular low-cost excited-state approaches were selected to assess their performance for core excitations. Of the wave function-based approaches, the CIS(D) and ADC(2) methods were chosen. The reliable performance of ADC(2) has been demonstrated in several excellent papers.^19−21,23,24,104^ Unfortunately, comprehensive studies on CIS(D) do not exist. Its usage is rather limited for core excitations; however, significant improvements have been realized compared with CIS and hybrid TDDFT results.^44,86^ Nevertheless, the accuracy of the methods for low-energy transitions is well-known. The performance of the ADC(2) method is outstanding for valence excitations,^16,105^ while the CIS(D) approach is slightly but consistently inferior compared with ADC(2).

In the case of the most simple DH functionals, spin-scaling techniques were not applied. One of the most popular of these methods is the empirically parametrized B2GPPLYP^106^ approach. Of course, such mixing factors can also be obtained using nonempirical considerations, such as the adiabatic connection formalism. The most successful nonempirical functionals are the PBE-QIDH^107^ and PBE0-2^108^ methods. The spin-scaling techniques enable higher flexibility of the energy functional; however, the number of parameters increases at the same time. One of the most widely used functionals in this class is DSD-PBEP86,^109^ where the mixing factors are parametrized for ground-state properties. In contrast, the spin-opposite-scaled variant of PBE0-2, namely SOS-PBE0-2,^110^ still contains only nonempirical parameters. The performance of DHs for core excitations is completely an unexplored field. Accordingly, at this point, we can comment on their accuracy only for valence electron excitations. As demonstrated in comprehensive benchmark studies,^83,111^ DSD-PBEP86 has excellent accuracy for valence excitations, while its error is significantly higher for Rydberg transitions. In addition, the overall performance of the nonempirical DH functionals is, surprisingly, better compared with the empirical B2GPPLYP approach. Furthermore, they provide a somewhat more balanced description of the corresponding excited states; however, their accuracies are not outstanding. To help the reader, the attributes of all of the functionals discussed in this subsection are collected in Table 1.

**Table 1:** Functionals Assessed in the Benchmark Calculations

functional	exchange	correlation	spin scaling	number of parameters	reference
PBE0-2	PBE	PBE	no	2	(108)
SOS-PBE0-2	PBE	PBE	yes	3	(110)
PBE-QIDH	PBE	PBE	no	2	(107)
DSD-PBEP86	PBE	P86	yes	4	(109)
B2GPPLYP	B88	LYP	no	2	(106)

## 4 Results

### 4.1 C *K*-Edge Excitations

**Figure 1:**
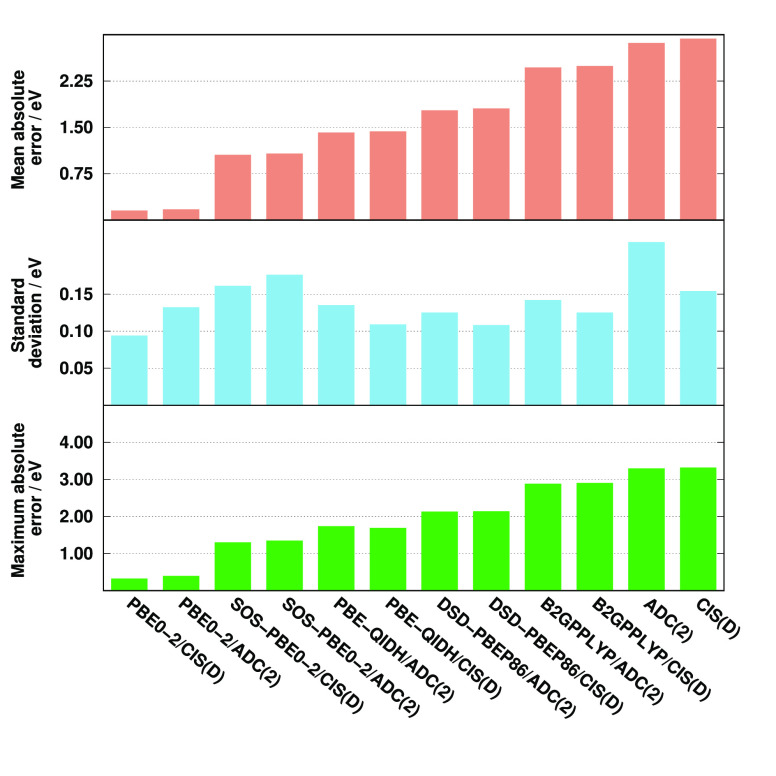
Error measures for the excitation energies of C *K*-edge transitions compared to EOM-CCSD references.

First, the results regarding the C *K*-edge excitations are discussed. The error measures obtained for the excitation energies are presented in Figure 1. Inspecting the MAEs, we can conclude that excellent performances are attained by the PBE0-2-based functionals. The errors are 0.15 and 0.17 eV for the CIS(D)- and ADC(2)-based approaches, respectively. Noticeably larger MAEs are obtained for the next functionals. That is, the errors are around 1.00 eV for the SOS-PBE0-2 approaches, while they are just below 1.50 eV for PBE-QIDH. Somewhat more unfavorable results are achieved by the remaining functionals; however, even larger MAEs are obtained for the wave function-based methods. The overall errors are 2.86 and 2.94 eV for the ADC(2) and CIS(D) approaches, respectively. Concerning the SDs, more balanced results are attained. The deviations are approximately 0.10 eV for the best performers, such as the CIS(D)-based PBE0-2, PBE-QIDH, and DSD-PBEP86 functionals. The SDs are still below 0.15 eV for the remaining approaches, except for SOS-PBE0-2 and the wave function-based methods. Again, consistent improvements can be realized compared with the wave function-based counterparts, especially for ADC(2). The same order can be determined among the approaches concerning the MAX errors as for the MAEs. Thus, outstanding results are attained by the PBE0-2 approaches, for which the MAXs are only about 0.35 eV, while the wave function-based methods are inferior, with MAXs of around 3.30 eV. Interestingly, highly similar MAEs and MAXs are obtained for the ADC(2)- and CIS(D)-based approaches, while the SDs are somewhat more favorable for the CIS(D)-based analogues.

It is instructive to scrutinize the results from a more chemical point of view as well. In this aspect, the largest and smallest errors are inspected to identify whether they came from a specific molecule or functional group. For the best performers, such as PBE0-2 and SOS-PBE0-2, the MAXs are obtained for the ethylene derivatives, for example, ethene, di- and trifluoroethene, and dichloroethene. For the remaining methods, the nucleobases, namely adenine, guanine, and cytosine, cause a significant problem. Interestingly, the lowest errors for these approaches are obtained for the ethylene derivatives. However, the absolute errors are still significant.

**Figure 2:**
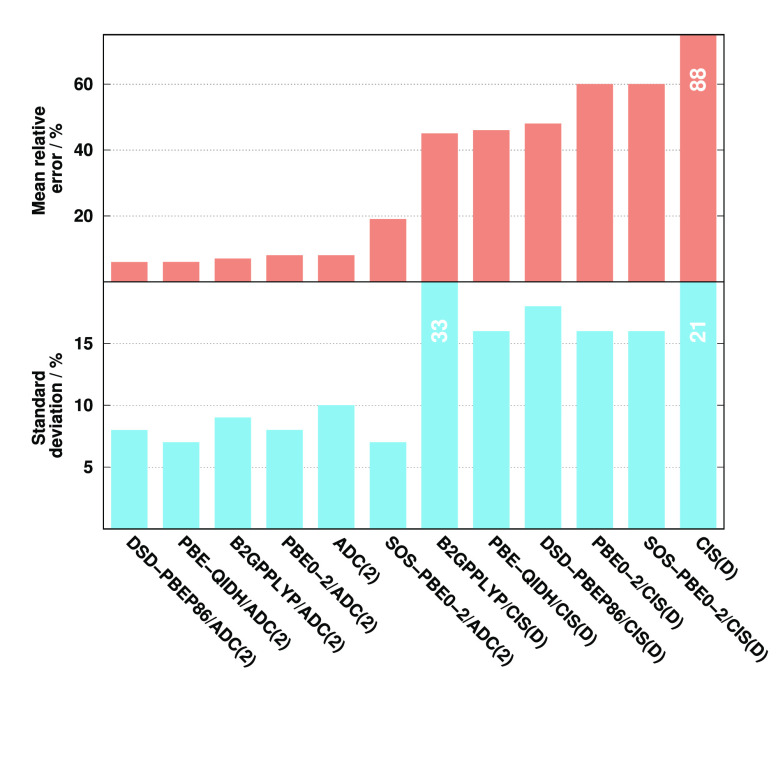
Error measures for the oscillator strengths of C *K*-edge transitions compared to EOM-CCSD references.

The error measures for the oscillator strengths are visualized in Figure 2. As can be seen, excellent and well-balanced performances are achieved by most of the ADC(2)-based approaches. That is, the MREs are between 6 and 8% for the best performers, while a significantly larger error, precisely 19%, is obtained for SOS-PBE0-2/ADC(2). Among the CIS(D)-based functionals, the most accurate results are achieved by the B2GPPLYP, PBE-QIDH, and DSD-PBEP86 approaches, with MREs of around 45%. The errors are just below 60% for PBE0-2 and its spin-scaled variant, while the CIS(D) method is inferior as its MRE is 88%. Thus, significant improvements can be realized for the CIS(D)-based DH functionals compared with CIS(D); however, they cannot compete with the ADC(2)-based analogues. Significant but milder differences can be observed for the SDs as well. The deviations fluctuate in a very small range for the ADC(2)-based functionals. The best precisions are measured for the PBE-based approaches, with a SD of about 7%, while it is still below 10% for B2GPPLYP/ADC(2). In contrast, concerning the CIS(D)-based methods, the lowest deviations are approximately 15%. This precision, again, is obtained by the PBE-based approaches, while the least favorable results are observed for B2GPPYLP/CIS(D) as its SD is 33%. The error measures have been evaluated for the most intense peaks (*f* > 0.05) as well. In this case, the MREs and SDs are somewhat more favorable; however, the improvements are highly consistent and insignificant. Therefore, the order of the performances hardly changes.

### 4.2 N *K*-Edge Excitations

**Figure 3:**
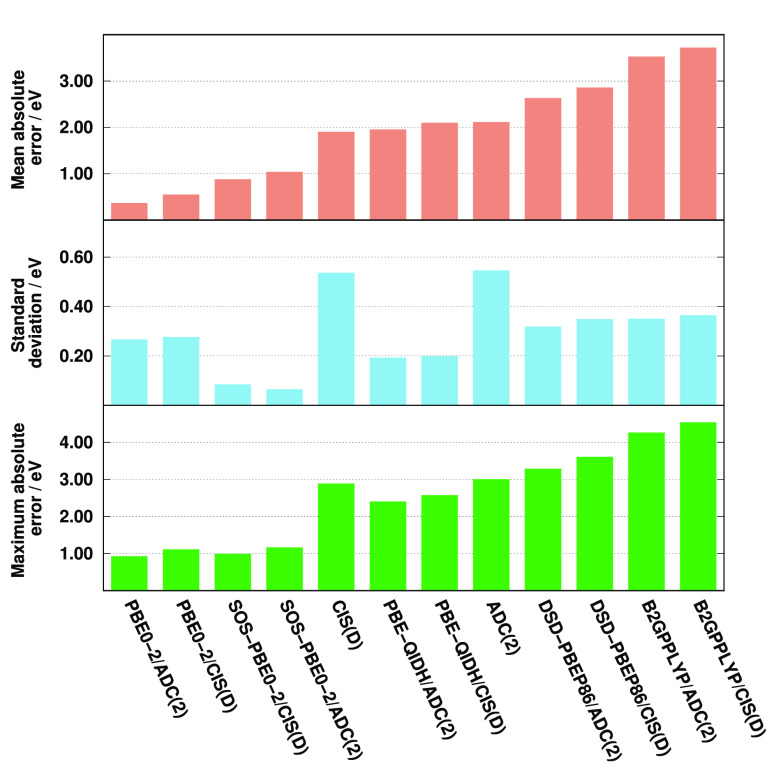
Error measures for the excitation energies of N *K*-edge transitions compared to EOM-CCSD references.

Next, the N *K*-edge excitations are assessed. The obtained error measures for the excitation energies are collected in Figure 3. The lowest MAEs are again obtained by the ADC(2)- and CIS(D)-based PBE0-2 approaches, with MAEs of 0.36 and 0.55 eV, respectively. The accuracy of the SOS variants is also outstanding, while a practically identical performance is measured for the PBE-QIDH- and wave function-based approaches. The errors are around 2.00 eV in these cases. The MAE starts to increase for the remaining methods. It is just below 3.00 eV for DSD-PBEP86, while the B2GPPLYP functional is inferior, with a MAE of about 3.50 eV. Inspecting the SDs, the performance of SOS-PBE0-2 is outstanding. The deviations do not exceed 0.10 eV in these cases, while they are still under 0.20 eV for the PBE-QIDH-based methods. The precision is also acceptable for the remaining functionals as the highest values, precisely 0.55 eV, are obtained for the wave function-based approaches. The same order can be determined among the approaches concerning the MAX errors as for the MAEs. The MAXs are around 1.00 eV for the genuine and spin-scaled PBE0-2-based approaches, while they are almost three times larger for the PBE-QIDH and wave function-based methods. Overall, we can conclude that the ADC(2)-based results are somewhat more favorable than the CIS(D)-based counterparts, except for SOS-PBE0-2; however, the differences are not significant at all. Inspecting the molecules and functional groups, we can conclude that the most significant errors are obtained for the nucleobases, except for SOS-PBE0-2, where these errors are extremely small.

**Figure 4:**
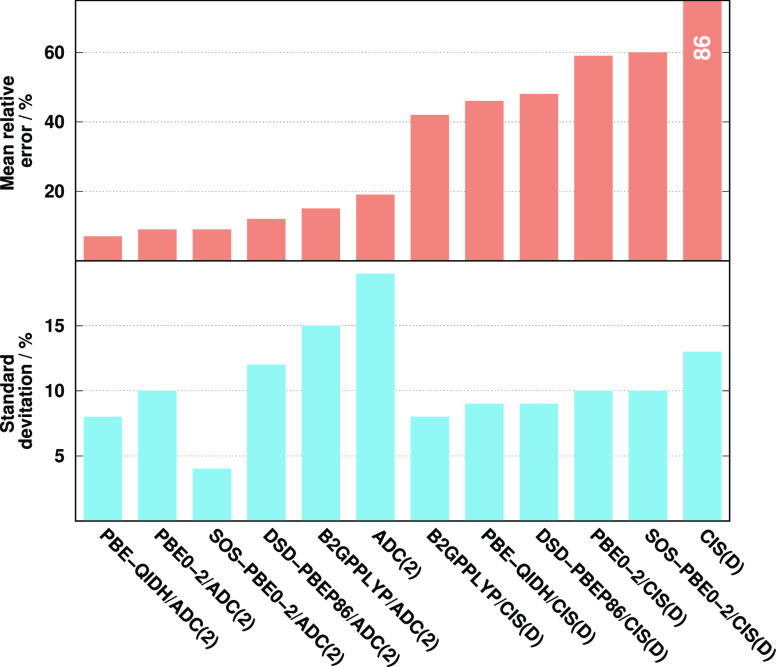
Error measures for the oscillator strengths of N *K*-edge transitions compared to EOM-CCSD references.

The overall performances regarding the oscillator strengths are presented in Figure 4. As can be seen, the lowest MREs by far are achieved by the ADC(2)-based approaches. The results are well-balanced for the PBE-based methods, where the MREs are below 10%. Somewhat notable errors are obtained for the remaining functionals; however, noticeable improvements are realized compared with ADC(2), where the MRE amounts to 19%. This difference is more significant for the CIS(D)-based approaches. In these cases, the CIS(D) method is inferior, with a MRE of 86%, while it is 60% for the least reliable DH functional. Surprisingly, in this class, the best performance is supplied by the B2GPPLYP/CIS(D) approach, where the MRE is 42%. It is six (three) times larger compared with the best (worst) ADC(2)-based DH. Similar to the excitation energies, the SOS-PBE0-2/ADC(2) method has an excellent precision, with a SD of 4%. The deviations are just below 10% for the ADC(2)-based PBE-QIDH and PBE0-2 approaches; however, it is also true for all of the CIS(D)-based DHs. Interestingly, the canonical CIS(D) results are more precise than the ADC(2) values, while the DHs always have lower SDs than the corresponding wave function-based counterpart. Inspecting the most intense transitions, most error measures are a bit more favorable, while the SDs are much smaller for the CIS(D)-based DHs.

### 4.3 O *K*-edge excitations

**Figure 5:**
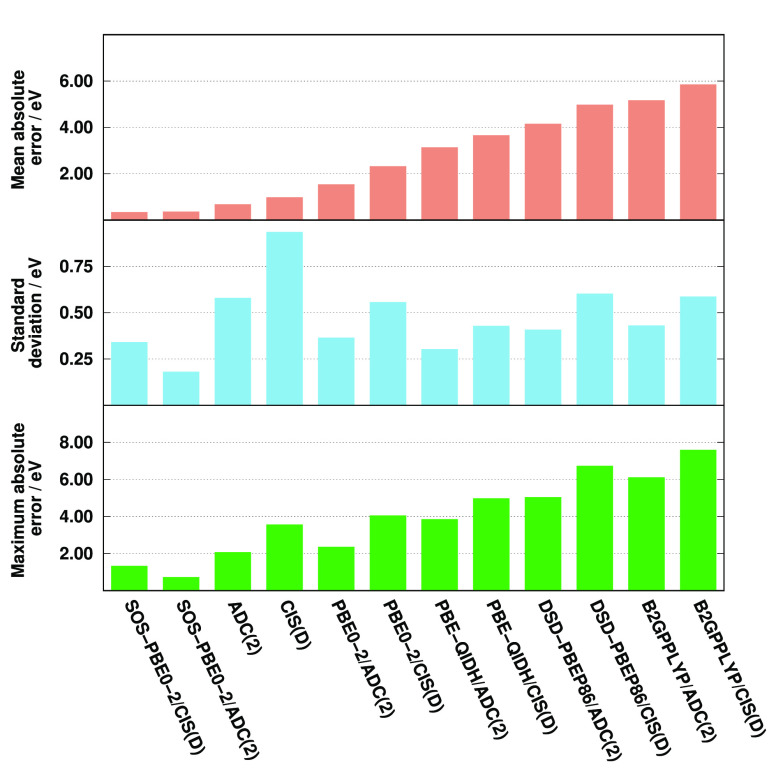
Error measures for the excitation energies of O *K*-edge transitions compared to EOM-CCSD references.

Finally, the O *K*-edge transitions are discussed. The corresponding error measures in the excitation energies are collected in Figure 5. On the basis of the numerical results, we can state that the most accurate excitation energies are attained by the SOS-PBE0-2 approaches, with MAEs of around 0.35 eV. Significantly higher but still acceptable errors are obtained by the wave function-based methods. In these cases, the MAEs are 0.67 and 0.97 eV for the ADC(2) and CIS(D) approaches, respectively. After this point, the error starts to increase rapidly. The MAE is just below 2.00 eV for PBE0-2/ADC(2), while it is already 2.31 eV for the CIS(D)-based counterpart. Even worse results are achieved by the remaining functionals; furthermore, the overall errors exceed 5.00 eV for B2GPPLYP. The precision of the methods is somewhat more balanced. The best performances are obtained by the ADC(2)-based approaches. The lowest SD, precisely 0.18 eV, is achieved by SOS-PBE0-2/ADC(2), while it is still under 0.40 eV for the PBE-QIDH/ADC(2) and PBE0-2/ADC(2) functionals. Inspecting the CIS(D)-based results, an outstanding precision, with a SD of 0.34 eV, is attained by SOS-PBE0-2. In addition, this measure is still moderate for the PBE-QIDH and PBE0-2 methods, while the deviations are around 0.60 eV for B2GPPLYP and DSD-PBEP86. Nevertheless, the CIS(D) approach is inferior as its SD is 0.94 eV. The lowest MAXs, precisely 0.72 and 1.33 eV, are provided by the ADC(2)- and CIS(D)-based SOS-PBE0-2 functionals, respectively. These values are 2.06 and 3.56 eV for the canonical ADC(2) and CIS(D) methods, respectively, while only the PBE0-2 functionals can compete with these results. In general, we can conclude that the error measures are more favorable for the ADC(2)-based approaches compared with the CIS(D)-based counterparts. In addition, the SDs are always lower for the DH functionals in comparison with the corresponding wave function-based method. However, the improvements in the MAEs and MAXs are not so consistent. Inspecting the molecules and functional groups, the most significant errors are still obtained for the nucleobases, while the SOS-PBE0-2 approach can safely be applied to these systems.

**Figure 6:**
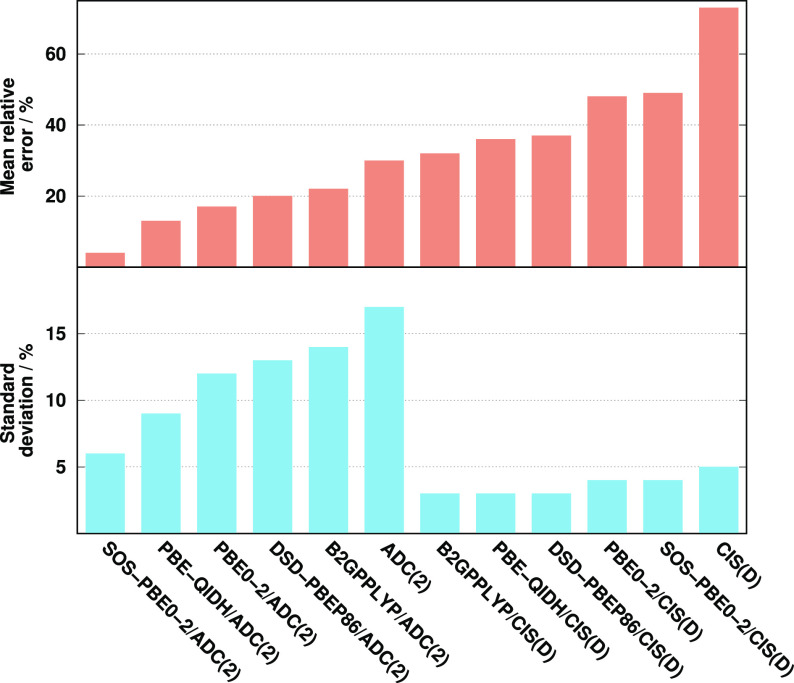
Error measures for the oscillator strengths of O *K*-edge transitions compared to EOM-CCSD references.

The error measures for the oscillator strengths are visualized in Figure 6. As can be seen, the MREs are less balanced compared to the previous results; nevertheless, all of the ADC(2)-based approaches outperform the CIS(D)-based methods. Similar to the excitation energies, the lowest error is attained by SOS-PBE0-2, with a MRE of 4%. This measure is 13 and 17% for the PBE-QIDH and PBE0-2 functionals, respectively. Among the ADC(2)-based approaches, the parent wave function method is inferior as its MRE is 30%. Concerning the CIS(D)-based approaches, the best performers are not so far from the ADC(2) method. That is, the MRE is still below 40% for the B2GPPLYP, PBE-QIDH, and DSD-PBEP86 functionals. Again, the least accurate approach is the wave function-based CIS(D), with a MRE of 73%, while the error is around 60% for the remaining DH functionals. Thus, significant improvements are realized for all of the DH methods compared with the corresponding wave function-based analogue. Inspecting the SDs, very surprising results show up. In this case, all of the CIS(D)-based approaches outperform the ADC(2)-based methods. The deviations are well-balanced for the best performers as they are still below 5% for CIS(D), which is the least favorable case in the CIS(D)-based class, whereas the highest precisions are provided by B2GPPLYP, DSD-PBEP86, and PBE-QIDH, with SDs of 3%. Significantly larger deviations are obtained for the ADC(2)-based functionals. The best performance is attained by SOS-PBE0-2, while the SD is just below 10% for PBE-QIDH. Again, the lowest precision, concretely 17%, is provided by the wave function method. Hence, despite the somewhat disappointing outcome, the DH formalism could improve the results. The picture somewhat changes when only the most intense transitions (*f* > 0.05) are considered. In this case, the SDs, being around 5%, are higher for the CIS(D)-based DHs, while it is around 8% for the ADC(2)-based ones. It means that the performances are more balanced in this case.

### 4.4 Overall Performance

To characterize the performance of the methods with a single measure, a procedure was recently proposed by Casanova-Páez and Goerigk.^111^ In the original study, the error measures were simply averaged for all of the benchmark sets assessed. We use the same measure in this study; however, the errors are averaged for the different types of *K*-edge excitations. We note that a somewhat different scheme was used in ref (24) to discuss the overall performance. The outcomes are mainly approached from two aspects: (i) the effects of the DH formalism are examined compared with the wave function-based results and (ii) the benefits of the ADC(2)-based formalism are inspected in comparison with the CIS(D)-based schemes. The numerical results are collected in Table 2. Using the EOM-CCSD results as reference, significant improvements are realized in the excitation energies for the PBE0-2 and SOS-PBE0-2 approaches. In these cases, the MAEs and SDs are noticeably lower compared with the corresponding wave function-based counterpart. Unfortunately, for the remaining functionals, the accuracies are not satisfying; however, the precisions are somewhat more favorable. Inspecting the excitation energies, notable differences cannot be observed between the ADC(2)- and CIS(D)-based formalisms. In contrast, the benefits of the former ansatz are demonstrated for the oscillator strengths. That is, the MREs are 11 and 56% for the ADC(2)- and CIS(D)-based (SOS-)PBE0-2 approaches, respectively. Interestingly, the precision of the CIS(D)-based DH functionals is also highly acceptable, with a deviation of 10%.

**Table 2:** Overall Performance of the Methods Using EOM-CCSD and ADC(2)-x Values As References^a^

	EOM-CCSD				ADC(2)-x			
	ω		*f*		ω		*f*	
method	MAE	SD	MRE	SD	MAE	SD	MRE	SD
ADC(2)	1.88	0.45	19	15	3.18	0.34	19	11
PBE0-2/ADC(2)	0.69	0.25	11	10	0.74	0.23	23	8
SOS-PBE0-2/ADC(2)	0.82	0.14	11	5	2.13	0.24	39	8
PBE-QIDH/ADC(2)	2.17	0.21	9	8	0.85	0.23	24	7
DSD-PBEP86/ADC(2)	2.85	0.28	13	11	1.54	0.22	15	7
B2GPPLYP/ADC(2)	3.72	0.31	15	13	2.40	0.24	13	8
CIS(D)	1.94	0.54	82	13	2.70	0.44	135	20
PBE0-2/CIS(D)	1.00	0.31	56	10	0.57	0.27	101	18
SOS-PBE0-2/CIS(D)	0.76	0.20	56	10	1.87	0.26	102	18
PBE-QIDH/CIS(D)	2.39	0.25	43	9	1.08	0.25	84	17
DSD-PBEP86/CIS(D)	3.21	0.35	44	10	1.90	0.27	86	17
B2GPPLYP/CIS(D)	4.02	0.36	40	15	2.70	0.28	80	24

a MAE(ω) and SD(ω) are given in eV, while MRE(*f*) and SD(*f*) are given in %. See text for detailed explanation.

The outcomes slightly differ when the ADC(2)-x values are considered as a reference. The PBE0-2 functional is superior in both classes, while the PBE-QIDH results are also outstanding in this comparison. Interestingly, all of the DH functionals outperform the corresponding wave function-based counterparts for the excitation energies. The improvements are more remarkable in accuracy, whereas they are somewhat milder but more balanced in precision. The gains are not so unequivocal for the oscillator strengths. Similar to the previous results, the improvements in the precision are consistent for all of the functionals, while the accuracies are significantly more favorable for the CIS(D)-based DH methods compared with the CIS(D) method. On the other hand, ADC(2) outperforms some of the ADC(2)-based DH approaches. Surprisingly, in this comparison, the lowest MRE is attained by B2GPPLYP/ADC(2), which was inferior to where EOM-CCSD results were considered as references. Nevertheless, the differences between the ADC(2)- and CIS(D)-based approaches are still remarkable.

## 5 Conclusions

In this study, DH theory has been successfully extended to core excitations. To this end, the working equations for the CIS(D) method utilizing the CVS approximation have been introduced. Thereafter, the concept of a genuine CVS-DH formalism was presented relying on the CVS-CIS(D) approach. In addition, a more advanced ansatz has been elaborated combining the CVS-ADC(2) method and our ADC(2)-based DH formalism.^78^ The resulting approaches were tested for the most popular DH functionals, such as the PBE0-2, SOS-PBE0-2, PBE-QIDH, DSD-PBEP86, and B2GPPLYP functionals. For the benchmark calculations, the recently proposed XABOOM^24^ test set was selected including 71 carbon, 21 nitrogen, and 22 oxygen *K*-edge transitions. Both accuracy and precision were comprehensively assessed for each method using fc-CVS-EOM-CCSD excitation energies and oscillator strengths as references, while the outcomes were briefly discussed against CVS-ADC(2)-x references as well.

The results were mainly discussed from two aspects. First, the benefits of the CVS-ADC(2)-based formalism were inspected in comparison with the CVS-CIS(D)-based one. Second, the effects of the CVS-DH formalism were assessed compared with the corresponding wave function-based approaches. Inspecting the excitation energies, our benchmark calculations show that the CVS-CIS(D)-based approaches are highly competitive with the more advanced CVS-ADC(2)-based methods. This finding is valid either accuracy or precision is considered. On the other hand, as expected, huge differences were observed in the oscillator strengths. In this case, the accuracy of the CVS-ADC(2)-based methods is significantly better; however, the deviations are acceptable for the CVS-CIS(D)-based functionals as well. Concerning the performance of the CVS-DH approaches, we can conclude that the most outstanding results compared to the fc-CVS-EOM-CCSD references are attained by PBE0-2, while its spin-opposite-scaled variant also seems to be reliable. The SOS-PBE0-2 functional could be an ideal cost-effective alternative concerning its robustness and computation time. For these approaches, significant improvements are realized compared with the corresponding wave function-based counterparts.

## Supporting Information Available

Error measures in the excitation energies and oscillator strengths for the C, N, and O *K*-edge transitions compared to EOM-CCSD and ADC(2)-x references, and excitation energies and oscillator strengths (XLSX)
